# AI-Enhanced Healthcare: Not a new Paradigm for Informed Consent

**DOI:** 10.1007/s11673-023-10320-0

**Published:** 2024-02-01

**Authors:** M. Pruski

**Affiliations:** 1 School of Health Sciences, The University of Manchester, Manchester, UK; 2 Department of Medical Physics and Clinical Engineering, Cardiff and Vale University Health Board, Cardiff, Wales UK

**Keywords:** Artificial intelligence, Patient consent, GDPR, Health informatics, Clinical informatics

## Abstract

With the increasing prevalence of artificial intelligence (AI) and other digital technologies in healthcare, the ethical debate surrounding their adoption is becoming more prominent. Here I consider the issue of gaining informed patient consent to AI-enhanced care from the vantage point of the United Kingdom’s National Health Service setting. I build my discussion around two claims from the World Health Organization: that healthcare services should not be denied to individuals who refuse AI-enhanced care and that there is no precedence to seeking patient consent to AI-enhanced care. I discus U.K. law relating to patient consent and the General Data Protection Regulation to show that current standards relating to patient consent are adequate for AI-enhanced care. I then suggest that in the future it may not be possible to guarantee patient access to non-AI-enhanced healthcare, in a similar way to how we do not offer patients manual alternatives to automated healthcare processes. Throughout my discussion I focus on the issues of patient choice and veracity in the patient–clinician relationship. Finally, I suggest that the best way to protect patients from potential harms associated with the introduction of AI to patient care is not via an overly burdensome patient consent process but via evaluation and regulation of AI technologies.

## Introduction

The use of artificial intelligence (AI) in healthcare has become a prominent topic in the healthcare literature in the last couple of years, due to the rise of such AI applications as medical image processing (McKinney, et al. 2020; De Fauw, et al. 2018; Davies, et al. 2022), clinical decision-making support systems (Sutton, et al. 2020), and support in managing medication (Zhao, et al. 2021). Consequently, the World Health Organization (WHO 2021) has published guidance on AI adoption in healthcare. Various aspects of AI’s clinical use have been discussed in the healthcare literature, and with reference to the General Data Protection Regulation (GDPR) the discussion on a patient’s right to an explanation of how AI makes its decision is of particular import (see, e.g., Astromskė, et al. 2021; Gerke, et al. 2020; Wachter, et al. 2017; Pagallo 2018; Selbst and Powles 2017; Hoeren and Niehoff 2018; Kim and Routledge 2018; Mourby, et al. 2021),^1^ though discussions of liability in cases of iatrogenic patient harm sustained during AI-enhanced care (Chan 2021; Schweikart 2020; Jobson, et al. 2022; Unver 2023; Kamensky 2020; Hodge 2021) are also related to the issue of consent, yet outside the scope of this paper. Here I focus on patient consent to the use of healthcare AI in making diagnostic decisions or therapeutic interventions. This is inclusive of AI applications in healthcare robotics where machines might both make decisions based on patients’ data and execute these decisions themselves. While it might still be some time until autonomous robotic surgery becomes reality (for a succint summary of the impact of AI on robotic surgery, see Hodge 2021, 421–422), this phenomenon of AI making clinical decisions is already manifested to some degree in intelligent patient ventilation software that automatically, within limits, adjusts the delivery parameters of mechanical ventilation, or by the MIRUS device which similarly adjusts the dosing of volatile sedatives (Bellgardt, et al. 2019). While other applications of healthcare AI exist, such as their use in highlighting patients at risk of deterioration, I will focus on the aforementioned areas, as diagnosis and treatment are the core activities we associate with medicine (Gamble and Pruski 2019). Moreover, the type of AI most relevant to my discussion will be machine learning—the ability of AI tools to learn from the data they have been trained on to provide a specific outcome (in our case, a diagnostic decision or treatment intervention). On this issue of AI, the WHO (2021) guidance makes two noteworthy statements:

1. “The use of machine-learning algorithms in diagnosis, prognosis and treatment plans should be incorporated into the process for informed and valid consent. Essential services should not be circumscribed or denied if an individual withholds consent” (26)
2. “There is, however, no precedent for seeking the consent of patients to use technologies for diagnosis or treatment. Nevertheless, the use of AI in medicine and failure to disclose its use could challenge the core of informed consent and wider public trust in health care.” (47)

I wish to challenge some aspects of these statements. I first introduce some of the fundamental legal cases that have shaped U.K. informed consent law and outline the pertinent aspects of the GDPR. These establish some clear cases where disclosure of the AI’s involvement must be made during the informed consent process, such as when there is no meaningful human input into a clinical decision made by an AI. I then highlight some scenarios that are not as clear-cut and which are based on currently available technologies and argue how more common adoption of AI technologies may further shape the consent process. I then provide a brief general discussion focusing on issues of privacy and regulation. Throughout this I highlight ethical issues which may seem to be quite novel but are actually well entrenched in the healthcare world, and consequently I propose that the introduction of AI into healthcare does not require any change in patient consent practice.

## Consent and the Law

Perhaps the most thorough discussion of patient consent to AI-enhanced healthcare is that of Cohen (2020). While Cohen conducts his discussion within the context of the United States, and as such it is not directly applicable to the United Kingdom, his arguments are persuasive and raise several important points for the purpose of our discussion. Cohen (2020, 1432–1434) notes that with respect to informed consent, two standards exist in the United States, physician-based and patient-based, which are applied dependently on the state one resides in. The physician-based standard requires the physician to disclose such risks as a reasonable physician would generally disclose to a patient. The patient-based standard requires that when discussing treatment, the physician should disclose such risks as a reasonable patient would expect to know in the given situation. In the United Kingdom, the latter standard is adopted, though it is somewhat broader as it also refers to the particular patient in question. This standard became enshrined via the *Montgomery v Lanarkshire* case ((Scotland) [2015] UKSC11), though guidance to this effect was already given beforehand by the General Medical Council (2008), with medical staff risking losing their licence to practise if they did not follow this guidance (General Medical Council 2019, 5). *Montgomery v Lanarkshire* states that the test is:

… whether, in the circumstances of the particular case, a reasonable person in the patient’s position would be likely to attach significance to the risk, or the doctor is or should reasonably be aware that the particular patient would be likely to attach significance to it. (Ibid)

The test makes reference to two standards: the subjective standard of the specific patient and objective standard of a reasonable person in the patient’s position. It also builds on the previous case of *Birch v University College London* ([2008] EWHC 2237 QB), which established that the patient has also the right to know of alternative interventions to the one advocated by the practitioner and their associated risks. Perhaps we may assume, as an example, that if most people in the United Kingdom would not want to have their surgery performed on them by a robot (PricewaterhouseCoopers (PwC) 2017), then that may set the standard of a “reasonable person.” Yet, such perceptions about robotic surgery may change with time. Cohen (2020, 1458), for example, doubts that there even now is a widespread “AI-phobia,” and a recent U.S. survey has shown a general favourable attitude of respondents towards healthcare AI (Dieter 2021). Nevertheless, *Montgomery v Lanarkshire* (Ibid) makes specific reference to the risks associated with the intervention, and it is doubtful whether simply being averse to AI implies that there is a specific risk associated with the procedure. Additionally, if a robotic AI performed, for example, a laparoscopic surgery with the same risks as a human surgeon, it is not clear if these should count as two different treatment options. So, with respect to *Montgomery v Lanarkshire*, it is not the presence of an AI (or robot) that constitutes an issue intrinsically but the presence of risk.

## AI and Claims of Battery

In the NHS a patient does not have a right to be treated by a specific doctor, though a patient in the United Kingdom undergoing private treatment may claim breach of contract if they had an agreement to be treated by surgeon X but were operated on by surgeon Y. This contrasts with the United States, where such a right exists and treatment by an AI-controlled robot as opposed to the doctor a patient expected may lead to a claim of battery (Cohen 2020, 1438–1439). Yet, patients in the United Kingdom still have a right to assume that if they consent for medical treatment, the treatment will be delivered by a qualified practitioner, such as a consultant surgeon and not just a technically competent person such as a trained vet; the vet may still be charged with battery even if the consultant surgeon let them do the operation and they carried it out perfectly (Brazier and Cave 2020, ¶5.5). Finally, it is important that the information given to the patient during the consent process be specific enough (Selinger 2009) for it to count as informed rather than just real consent; failure to obtain real consent can raise claims of battery while failures to obtain informed consent can give rise to claims of negligence (see *Chatterton v Gerson* [1981] QB 432). As I will discuss later, healthcare AI and medical devices may well be given a status that justifies their use in patient care without the need to obtain explicit consent from a patient for their use. This is crucial as otherwise this opens the possibility of claims of battery if the patient is operated on using technology to which they did not explicitly consent.

It is important to consider scenarios outside of surgery since the *Montgomery v Lanarkshire*case explicitly states the above conditions for valid informed consent with reference to treatment:

The doctor is therefore under a duty to take reasonable care to ensure that the patient is aware of any material risks involved in any recommended treatment, and of any reasonable alternative or variant treatments. (Ibid)

As such, let us see if these consent considerations apply in other contexts. Here the case of Gallardo v Imperial College Healthcare NHS Trust is important ([2017] EWHC 3147 QB, ¶73, 75, 84), as it highlights that the care team should provide the patient with the necessary information at all stages of care, though that specific case focused on post-treatment discussions. *Gallardo v Imperial College Healthcare NHS Trust* shows that there are risks to the patient that relate to information non-disclosure, such as the possibility of the recurrence of a cancer. Yet, the whole debate cannot be reduced to just material risks. As the judge noted in ¶80, a doctor who does not communicate well “risks losing the patient’s trust and confidence, and the patient’s right to be informed is not respected.”

The law highlights that the key element of the informed consent process is the communication of risks. As such, it seems that whether any special disclosure of the role of AI in a particular treatment or investigation has to be made will depend on the AI associated risks. There are, though, further considerations relating to honestly communicating with the patient about who will take care of them and how during the procedure or investigation, that is, considerations of maintaining trust in the patient–clinician encounter. As such, the patient does seem to have a right to be informed about the role of AI in their care, at least in some circumstances, based on the legal precedent in the United Kingdom, despite the WHO claiming (WHO 2021, 47) that precedence for this does not exist.

## AI Related Legislation

In the United Kingdom and in the European Union, the most pertinent piece of legislation to the use of AI, including in healthcare, is the GDPR.^2^ The purpose of GDPR is to protect the fundamental freedoms of citizens with respect to how their data is being controlled and processed by third parties (Room 2021). The GDPR protects the personal data of natural persons within the regions that enacted this legislation^3^ but also when the data of these natural persons is processed outside of the borders of these geographical regions (see art. 1(2), 2, 3(1,2,3) of the GDPR). “Processing” covers both manual and automated means of performing operations on personal data and includes the following in its definition: collection, structuring, storage, and use (GDPR article 4(2)). Moreover, GDPR (in article 4(7), (8)) recognizes that processing and controlling of data can be undertaken by a natural or legal person, a public authority, agency, or other bodies. As such, GDPR applies in a wide variety of situations.

The definition of healthcare data (“data concerning health”) is given in article 4(15) of the GDPR as “personal data related to the physical or mental health of a natural person, including the provision of healthcare services, which reveal information about his or her health status.” Moreover, article 9(2) of the GDPR specifies that alongside biometric and genetic data, processing of healthcare data is prohibited and lists ten exceptions to this prohibition, including:

(a) “the data subject has given explicit consent [...] for one or more specified purposes”

(h) “processing is necessary for the purposes of preventive or occupational medicine, […] medical diagnosis, the provision of health or social care or treatment or the management of health or social care systems and services on the basis of domestic law [in the U.K.] or pursuant to contract with a health professional”

(i) “processing is necessary for reasons of public interest in the area of public health”

(j) “for archiving purposes in the public interest, scientific or historical research purposes or statistical purposes”

The GDPR also specifies various requirements relating to the *automated* processing of data, that is, that which occurs without human involvement. It states in articles 13 (2, particularly point f), 14 (2, particularly point g), and 15 (1, particularly point h) that data subjects must be informed about automated decision-making relating to their data, including the logic involved in the processing and the envisaged consequences relating to this processing, and (as in cases not involving automated processing) they must be granted access to that data, as well as information about who will have access to their data, how long it will be stored for, and for what it will be used for—this holds true whether the data was obtained from the subject or not. Importantly, the way the legislation is phrased makes it explicit that it applies to analysing and predicting a person’s health. Article 22(1) of the GDPR then grants persons a “right not to be subject to a decision based solely on automated processing, including profiling, which produces legal effects concerning him or her or similarly significantly affects him or her.” As such, even if processing is permitted under the exceptions listed in article 9 of the GDPR, article 22 limits the applicability of automated processing in cases that fall within the scope of these article 9 exceptions if they could significantly affect the patient, such as cancer misdiagnosis may. Importantly, while article 22(2) of the GDPR grants some exceptions to this prohibition, these exceptions only apply to healthcare data (as well as genetic and biometric data) if the data subject gave their consent to such automation or there is substantial public interest for doing so and appropriate safeguards are put in place (see article 22(4) of the GDPR). Additional information relating to this matter is provided in recital 71 of the GDPR, including a “right […] to obtain an explanation of the decision reached after such assessment and to challenge the decision” (it is noteworthy that recital 71 also states that automated processing “should not concern a child”). Finally, GDPR also specifies the conditions for valid consent necessary for lawful processing of data in many situations in articles 4, 7, and 8. With article 4(11) specifying that consent has to be “freely given, specific, informed and unambiguous.”

Importantly, there has been ongoing debate as to how this regulation applies to the healthcare setting in relation to matters of patient consent in the context of AI (Astromskė, et al. 2021; Gerke, et al. 2020; Mourby, et al. 2021). Some scholars highlight that recital 71 is not legally binding (since it is a recital rather than an article of the legislation), and hence there is no legally binding right to explanation of individual decisions (Wachter, et al. 2017). However, whatever the implication of the binding status of recital 71 of the GDPR is (e.g., courts sometimes use recitals to clarify the meaning of the law (Pagallo 2018)), articles 13(2)(f), 14(2)(g), and 15(1)(h) of the GDPR clearly provide a right to “meaningful information about the logic involved, as well as the significance and the envisaged consequences” (Selbst and Powles 2017), while article 22 of the GDPR grants a right not to be subjected to automated decision-making. As such, it seems that in cases of autonomous AI systems (i.e., those which make decisions without meaningful human input), whether diagnostic or treatment providing, patients may need to explicitly give their consent for their use (see Hoeren and Niehoff 2018 in the context of a right to explanation). Indeed, Mourby, et al. (2021, 4–6) suggest that because of this high bar presented by article 22 of the GDPR, the use of systems that make “solely automated decisions” will be rare, as the patient would in these situations have a right to meaningful information about how a potentially black-box AI makes the decision. This may be a barrier to gaining consent within the scope of GDPR if the clinician cannot provide such an explanation and implies great challenges in obtaining adequate patient consent under GDPR (Mourby, et al. 2021,5) when using such solely automated decision systems. Nevertheless, what the critique provided by Wachter, et al. (2017) of the meaning of such a right to explanation and Selbst and Powles’s (2017) discussion of Wachter, et al. (2017) highlight is that this right to explanation may well be limited to only a broad generic explanation of how the AI works (rather than how it made a decision in the case of a specific patient), which is more feasible to achieve in a clinical context and probably more desirable; though Mourby, et al. (2021) reached their aforementioned conclusions based on similar assumptions of the level of information necessary to be given during the consent process as Selbst and Powles (2017) assumed. Additionally, while the right to challenge any such automated decision is described only in recital 71 of the GDPR, article 21 provides broad rights to object to the use of automated decision-making, suggesting that patients in principle may object to the use of such AI systems in their care.

The reviewed consent law has highlighted the need to state any risks associated with a healthcare intervention that the patient may judge material; the GDPR has highlighted the need for transparency about the involvement of AI. For example, if a healthcare diagnosis was made based solely on automated AI processing, then patients have more specific rights to know how the AI made its decision and to opt out of this AI-based decision-making. If the processing of the data is not automated, then the processing of healthcare data falls within the scope of the exceptions listed in article 9. This means that such use of healthcare AI may not require disclosure if it was not associated with risks material to the patient—at least in those cases when clinicians use AI systems to support the diagnosis rather than to replace their diagnostic deliberations (i.e., where human input is “meaningful” and not just a “nominal” “token gesture”; Hoeren and Niehoff 2018, 312). Mourby, et al. (2021, 7 and 13), however, argue (largely based on recital 60 of the GDPR and the deliberations of the Article 29 Data Protection Working Party 2018) that such a disclosure should still occur when clinicians make meaningful input into AI-aided decisions. I will comment on the reasonability of GDPR regulation in this context further in the article. To see how this may affect clinical practice, let us turn to some examples.

## AI versus “Traditional” Practice

So far, we have established that mandatory disclosure of the use of AI is largely limited to those situations involving either the type of AI-executed robotic surgery as portrayed in Prometheus (Scott 2012) or AI-automated diagnostics as per the holographic Doctor in Star Trek Voyager (Berman, et al. 2000). While some of the clinical applications of AI are moving from the realm of science fiction into the real world, present-day practice does (at least by and large) not meet the threshold for obligatory up-front disclosure. Therefore, below I briefly discuss two cases where AI may enhance care but does not act autonomously to a degree where the above-mentioned thresholds would be clearly crossed. In these cases, meaningful human input is preserved and there is no additional risk from the AI input. I do this to consider whether while such use of AI may not need to be explicitly disclosed according to legislation and common law; perhaps there may be some other underlying reason to do so, and the law may not be currently fit for purpose.

Firstly, consider a case similar to that discussed by Schiff and Borenstein (2019) of a surgical guidance system, as well as the general advances of surgical robotics exhibited by the Da Vinci (Da Vinci Surgery n.d.) and Versius (CMR Surgical n.d.) assistive surgical systems. These systems are beyond the scope of GDPR as their use requires meaningful human input in their operation. They consist of robotic arms controlled remotely by surgeons, allowing them to execute laparoscopic-like surgery, such as prostatectomies (Thomas, et al. 2021; Crew 2020), while sitting at a console. When giving consent, patients consent to a specific type of surgery such as laparoscopic surgery or open surgery, and perhaps some may regard this distinction between types of surgery as a relevant analogy to the difference between AI-enhanced and non-enhanced surgery. Presumably, the reason for gaining consent to a specific type of surgery is that the potential side effects from open surgery are greater than from laparoscopic surgery. Since one ought to seek patient consent specifically for an open surgery, let us now consider if this means that one must also do it for an AI-assisted surgery.

To recap, we have already established that a patient should be given information pertaining to risk, as established by the *Montgomery v Lanarkshire* case, while *Chatterton v Gerson* talks about proceedings that can be brought against hospital personel if the relelvant information is not disclosed to the patient, such as who will be operating on them. Now, considering that, in the case of prostatectomies, the risk associated with current robotic surgery is similar, if not slightly lower, than that of traditional laparoscopic surgery (Crew 2020). Therefore, it seems that the presence of the robotic system may not in principle require a specific patient disclosure due to the risks involved. Moreover, since the surgeon retains the practical responsibility for the operation, no claims of battery should arise.

Yet, one may argue that an assistive-AI used with meaningful human input may still make a mistake and hence the use of the AI should be disclosed. This statement can nevertheless be countered by the fact that the surgeon may also make a mistake during a traditional surgery. Moreover, in the United Kingdom, as long as the technology is fit for purpose, a specific need for the disclosure of its use is superfluous, at least within the context to which the patient has agreed. A disclosure may need to be made if the use of a different technology was associated with different risks, such as how the use of ionizing versus non-ionizing radiation during a procedure is associated with different risks of cancer. This is reflected by the fact that surgeons do not obtain consent from patients for the use of specific instruments during the surgery, since patient trust that surgeons will use those tools which will make their job better.^4^ To further illustrate this point, consider briefly the case of intraoperative patient monitoring by the anaesthetist as a supplementary aspect to our surgical deliberations. The anaesthetist does not declare to the patient all the modes of monitoring they might employ during surgery. If the anaesthetist would want to monitor the patient’s cardiac output, there is a wide variety of systems that they can employ to this aim, such as calibrated and non-calibrated systems utilizing the patient’s arterial and central venous lines and using a variety of algorithms to calculate the values of interest, as well as echocardiography methodology. As such, at least when the input of the AI is relatively small and not associated with an increased procedural risk, routine disclosure of an AI’s involvement seems not to be warranted since it is not different from the surgeon or anaesthetist using the tools they professionally deem best in a given situation.

Let us now consider if the patient should be informed of different options available to them, as consequent to the *Birch v University College London* ruling. If the robotic surgery is treated as laparoscopic surgery, merely executed via the robot as tool rather than via traditional equipment, then the alternative to robotic surgery is not non-robotic surgery, but open surgery, especially if robotic surgery becomes commonplace or even the standard of care. Now consider that in the future the robot will be able to carry out the operation itself, with the surgeon just supervising it in case it malfunctions. In this case the surgeon will have no meaningful input into the actions of the robot. The robot analyses the visual and physiological information itself, and executes the therapeutic actions of the surgery without human input. This may make the robot’s actions fall within the scope of the GDPR. Now assume that the robot performs prostatectomies as well as a human surgeon can, or perhaps even slightly better, and such surgery becomes commonplace. I will soon discuss whether there are important differences between the present state and this potential future state.

Now consider any of a plethora of AI systems that help with diagnosis. These systems are becoming more common, especially in the area of image analysis, where some can outperform their human counterparts at achieving the correct diagnosis (McKinney, et al. 2020; De Fauw, et al. 2018; Davies, et al. 2022). AI systems may also provide help in other diagnostic areas such as electrocardiogram result interpretation (Siontis, et al. 2021). Now consider that a physician is using such an AI as an adjunct rather than a replacement for making their diagnosis. It may be argued that patients should be informed that an AI is being used as a tool in their diagnosis provision since it is associated with various problems. The AI may not have been trained and tested on an ethnically appropriate data set or there may be another bias in the data used for the AI’s development (Norori, et al. 2021; Ibrahim, et al. 2021). There may be concerns about the AI’s black-box nature, which cannot “explain” its reasoning. Yet, ostensibly these are not new challenges in healthcare, and while this does not mean that we should not attend to them, it does mean that there seems to be little warrant to treat AI-enhanced care differently to normal care because of the existence of these problems.

Issues of lack of replicability, small sample sizes, or sample populations not being inclusive enough are frequently being reported in the scientific media, with perhaps the most seminal work highlighting these problems with respect to pharmaceutical research being that of Goldacre (2012). Moreover, biases in training may be as prevalent in AI training as in human clinical training. This has, for example, been the case with the assessment of dermatology conditions in patients with darker skin (Ibrahim, et al. 2021; Mukwende, et al. n.d.; Kamulegeya, et al. 2019). As such, it cannot be argued that the occurrence of these problems in healthcare AI warrants a more stringent consent process.

AI explainability may be desirable for reasons beyond fulfilling GDPR requirements, since it may potentially advance science by revealing causal relationships that will allow us to further improve healthcare, though it is uncertain if this will happen in practice. Yet, what is more relevant from a clinical perspective is AI reliability (London 2019). In reference to this, Cohen (2020, 1459) draws an analogy with a junior doctor asking a senior for advice. If the junior has good grounds for trusting the senior’s opinion more than their own, the junior is still acting in the patient’s interest by following the senior’s advice, even if the junior does not fully comprehend the senior’s explanation. A similar situation might occur when a generalist, such as a general practitioner or an intensivist, acts on the advice of a specialist, such as a haematologist. Of course, it is easier to scrutinize the AI if we know the “reasoning” it followed, but even if we do not understand it, it may give the doctor an important alternative consideration. This point about reliability being more important than explainability is also highlighted by the fact that even in recent history we were not able to reliably explain how commonly used medications worked despite having good evidence that they did (Harmer and Cowen 2013; Emmanouil and Quock 2007; Graham and Scott 2005). Of course, this does not mean that explainability is unimportant but that the issue of reliability is more central.

Seemingly, there is little new with respect to risk in relation to patient consent that present-day AI healthcare technologies bring forward. As such, while GDPR creates larger demands with regards to the patient consent process when there is autonomous decision-making by an AI, such demands do not seem justified if the AI technology is safely implemented. Of course, this does not mean that we should be satisfied with AI technologies making mistakes or being based on poorly conducted research. Indeed, perhaps having medical AI in the spotlight offers us a chance to correct these problems in medicine. But I would argue that the consent process is not the place to do this, especially since patients often do not have the technical know-how to effectively interrogate the AI for the presence of such problems. I will later discuss why the best safeguard against this is a robust evaluation framework and a system for raising concerns. Yet first we must also consider the patient’s perspective on this issue and place it in the context of ethical frameworks, and then discuss the potential impact of healthcare AI technologies becoming ubiquitous.

## Patient Considerations

While the legislation, common law, and guidance from professional bodies are key to delimitating the requirements of healthcare professionals with respect to obtaining a patient’s informed consent, there is more to the therapeutic relationship than just legal tick-boxes. For example, when *Montgomery v Lanarkshire* refers to a “reasonable person” and “material risks,” it is impossible to understand what these are without reference to the mores of society. Moreover, as *Gallardo v Imperial College Healthcare NHS Trust* states, there in an important aspect of maintaining patient trust and confidence in the therapeutic process, and the law provides no simple instruction how to achieve this.

While no ethical framework gives easy answers to the issue of information disclosure, some principles can help us to tease out relevant considerations. The principlist principle of autonomy and virtue of veracity (Beauchamp and Childress 2013) are particularly relevant to the issue of informed consent (Lorenzini, et al. 2022; Schmitz-Luhn, et al. 2022). Western attitudes towards the therapeutic relationship have somewhat changed in the last decades, with patients taking a more active role in decision-making rather than deferring to the opinions of clinicians; a shift from a paternalistic medicine model to one of patient autonomy (Kilbride and Joffe 2018). While this is not to say that patients ignore the opinion of medical staff, patients make decisions about their treatments that reflect their own values (the subjective standard in *Montgomery v Lanarkshire*), as for example in the case of vaccinations (Pruski 2021), and are not only reliant on clinicians’ preferences, whether these be due to the effectiveness of a treatment or the clinicians’ own values. This means that patients are free to make clinically unwise decisions (British Medical Association 2019).

As such, in the wake of recent development around ChatGPT (Future of Life Institute 2023), some patients may simply refuse AI involvement in their care because they fear that it will turn them into the Borg (a fictional cyborg faction from the Star Trek franchise; see for example Berman, et al. 2000) or because the patient’s own ethical principles motivate them to make choices that increase the need for human employment and they fear that permissive attitudes to AI will decrease employment opportunities for humans. These two latter worries may not be considered material risks by the objective standard in *Montgomery v Lanarkshire*, but patient decisions based on these beliefs still have to be respected, unless issues of mental capacity come into play.

## Veracity

I will now consider some ethical principles with respect to the conduct of individual clinicians. As the issue of disclosure of the role of AI in a patient’s healthcare journey is at the heart of my deliberation, I will focus this discussion on the virtue of veracity. Veracity, which can also be described as truthfulness and honesty, plays a key role in facilitating patient choice, which I highlighted as a very important phenomenon in modern Western healthcare and one that is at the core of informed consent legislation.

If the aforementioned risks of introducing AI to patient care are not considered material, or perhaps even reasonable by the majority of people, but are still relevant reasons for a specific patient’s decision-making, then the virtue of veracity should still motivate the clinician to disclose the involvement of the AI. Clinicians should truthfully answer any questions a patient might ask. The difficulty here is that a patient may not even consider that AI would be involved in their care, so even if they have an aversion to AI, they might not think to ask about its potential involvement. Moreover, the clinician may not be reasonably aware that the patient places significance to it, so might not mention it, or perhaps the clinician may be somewhat oblivious to the input of the AI because they do not fully understand the inner workings of all the technologies they use. Perhaps, to be on the safe side, clinicians should always disclose the involvement of an AI? Yet, this may not seem congruent with the principle of disclosing material risk judged as such by the subjective and objective standard of *Montgomery v Lanarkshire* and may not represent the best use of sparse healthcare professional time (Iqbal and Christen 2022) in all situations,^5^ especially if AI involvement becomes commonplace.

Of course, it may be the case that some patients who are averse to AI might know that their aversion is to some degree irrational and would rather simply not know of the AI’s involvement to avoid worrying about AI involvement while being able to reap the benefits of the technology. This presents a difficulty in that a clinician cannot be aware that a patient would rather not know about AI’s involvement unless a patient stated this before the healthcare professional mentioned AI themselves. The alternative is to invoke therapeutic privilege—that is, nondisclosure for the patient’s benefit—regarding the involvement of AI in a patient’s care (Nolan 2023), but the assertion of therapeutic privilege should be an exception, rather than the norm. Moreover, an opportunity to converse about this topic may alleviate patient fears if they originate from, for example, a misunderstanding of the degree of automation that is involved in an AI-enhanced healthcare intervention (Stai et al. 2020). They also can be an opportunity to address unrealistic patient expectations of AI performance (Ursin, et al. 2021).

But such conversations cannot happen if fidelity to trust (Pellegrino and Thomasma 1993) is not established and there is no transparency in the behaviour of the healthcare professional (Lorenzini, et al. 2022; Kiseleva, et al. 2022). When these conversations do happen, it is important to remember that they are there to facilitate shared decision-making (Lorenzini, et al. 2022). Ursin and colleagues provide a simple guide as to what should be included in such a consent discussion and why (Ursin, et al. 2021), but the way things are explained should be geared to the understanding of the patient, and there may be good reasons to limiting the number of points covered (Iqbal and Christen 2022).

The ethical principles discussed here show that there is more to informed consent than just simple considerations of material risk, as other factors may legitimately affect patient choice. Moreover, with a variety of attitudes towards technology in society, it is hard to pinpoint what information a reasonable person expects with respect to AI involvement in their care. Until case law manages to better dissect this issue or until major involvement of healthcare AI becomes commonplace,^6^ prudence may suggest informing patients upfront about the role of AI if it has a substantial input into the diagnostic or therapeutic process, as in the case study presented by Iqbal and Christen (2022).

## What if AI Becomes Ubiquitous?

While I have shown that legislation and common law can in certain circumstances mandate the disclosure of the use of AI in healthcare, I have also argued that situations in which such a disclosure becomes mandatory according to current legislation may become more common in the future. Now I wish to briefly argue that if such AI-enhanced care becomes common, the requirement to specifically request consent for AI-enhanced care will become bizarre by current clinical standards.

Consider an AI technology that, in the hands of a healthcare professional who is not trained to diagnose deep vein thrombosis (DVT), makes DVT diagnostic decisions (ThinkSono n.d.). Here an automated decision as defined by GDPR is clearly made since human involvement is only nominal (Hoeren and Niehoff 2018). But consider now, as a thought experiment, that the National Institute for Health and Care Excellence (NICE) had found, when evaluating the technology, that the use of such a technology in emergency departments is associated with better patient outcomes and lower costs. In other words, human care alone was inferior to AI-enhanced care. In this situation, it may appear odd to demand explicit disclosure of the AI’s use during the patient consent process, since by the *Montgomery v Lanarkshire* standard it is the risk of human care alone that should be primarily highlighted.

But now consider that, in the case of the above scenario, a disclosure is made and the patient requests the AI not be used but still wants to be treated. While this may seem understandable at face value, this is analogous to the case of someone coming to the emergency department refusing best available medical treatment and instead asking for bloodletting because they trust treatments with a well-established past. The law is clear that the doctor cannot be forced to provide care they deem to be not compatible with their professional judgement even by the courts (Re J (A Minor) (Wardship: Medical Treatment) [1991] 1 Fam 33) and perhaps the patient might not find a doctor willing to provide such care because the risks of practising without AI may not survive professional peer scrutiny (Cohen 2020). It is the patient’s prerogative to choose not to receive such AI-enhanced care, but it seems impossible to always guarantee, as the WHO guidance stipulates, that a non-AI-enhanced alternative will be available (World Health Organization 2021); to make reference to *Birch v University College London*, there might not be any immediate clinical alternative. While in principle the doctor should arrange for the patient’s transfer to another doctor who would be willing to provide such care (Ms B v An NHS Hospital Trust [2002] EWHC 429 (Fam)), there simply might not be any who would provide it. If such an alternative is not available, then “consent as a basis for processing under the GDPR” (rather than informed consent to treatment) may not even be possible, as the patient cannot consent without the threat of not receiving care if they refuse the AI-enhanced intervention, and so the consent “cannot be ‘freely’ given” (Mourby, et al. 2021).

As such, it would be somehow odd, if AI became the standard of care for improving clinical decision-making, for legislation to demand a disclosure of AI’s use explicitly in the consent process. This does not mean that a disclosure should not be made if the patient explicitly asks about AI involvement (a doctor, or any healthcare professional for that matter, has a duty to answer questions truthfully) or that a refusal to AI-enhanced care should not be respected. But in the future, it may be similarly odd for that to happen as it would be now for a patient to ask if automated analysers were used in the analysis of their blood samples, then demand that these should not be used, with the patient only accepting care if the Biomedical Scientists analysed the samples manually. It may simply become impractical to provide care that is not AI-enhanced in the future; to return to the previously mentioned consideration of robotic surgery with only token human input, it may be too inefficient to offer only human-provided care and to justify the right of patients not to have AI involved in their care. While the aforementioned ethical considerations may support a patient’s right to object to AI care in the future, utilitarian considerations of cost-effectiveness may stop us from offering a patient an alternative, at least in some situations.

As the blood sample analysis example shows, we do not judge the presence of automation as shifting the category to which a specific procedure belongs, it is still a blood sample analysis; similarly in the case of robotic surgery, neither should the presence of an AI-enhanced robot shift the laparoscopic surgery into a new category. The patient who has blood taken for analysis consents to the analysis and trusts that it is being done using an appropriate methodology. The thing that ensures that this consent process is appropriate is not that we tell the patient that the analysis is done via an automated analyser rather than a human or give them details about the technical functioning of the analyser (we do not do either of these), but it is ensured by accrediting the analytical labs themselves, licensing the people who work in them, and regulating the equipment that is used by them. Having similar process for healthcare AI is what may ensure that they maintain medical standards, which lies at the core of the issue of the use of these technologies (Schmitz-Luhn, et al. 2022).

## Brief Recap

In the United Kingdom, there seems to be relevant legal precedent and AI-specific legislation relating to the issue of informed consent when AI-enhanced healthcare is used, contra the second quote from WHO (2021) that I gave in the introduction. Moreover, I have argued above that at least in some cases it may be superfluous to disclose the involvement of an AI system in the care process if there is no ground to believe that the patient holds this as a materially significant factor in the consent process. Nevertheless, I acknowledge that since the use of AI is still sparse, we should perhaps not expect patients to be up-front with such concerns. As such, it might for the moment be more prudent to be explicit about the AI’s involvement. This will change as AI becomes more ubiquitous in healthcare.

## Data Privacy

Some may argue that the consent consideration is significantly changed by the fact that the AI might learn from the data it obtained during the treatment of patients. This is true if the data are being used commercially or for research purposes. Yet if neither of those cases apply, but the data are used purely for the AI to improve its performance within the institution (i.e., to continuously learn), then this is analogous to a healthcare professional becoming more experienced and learning from their day-to-day practice or a department learning from an internal audit. Of course, the institution may still need to pay licence fees for such an AI system if it was not developed in-house, and its manufacturer will in some sense benefit financially from such learning of its AI programme even if this learning is confined only to that particular institution. Yet, the same would hold true if a hospital was paying an agency that supplied staff (for example doctors to cover extra shifts) and these staff would also gather more experience and become better, which may potentially encourage the hospital to continue paying this agency for the supply of these staff members. So at least in this case, privacy should not be an issue.

The bigger issue with respect to privacy seems to be data security. Even recently, poor levels of digital security were reported among European hospitals, and there is no reliable standard for the privacy assessment of digital applications (Benjumea, et al. 2020; Uwizeyemungu, et al. 2019). Since privacy issues do not uniquely affect AI, I do not believe that privacy considerations force AI-enhanced healthcare consent into a new category. The use of digital healthcare systems that process sensitive data is common even without AI involvement; examples of such use include patient record management systems and applications for online healthcare consultations. This simply highlights data security as a key area for improvement in medical practice generally, in a similar way to how the use of unrepresentative patient cohorts in clinical studies affects both AI technologies and more traditional clinical approaches to care. Not addressing this issue can result in clinically useful AI being sabotaged for nefarious purposes (Schmitz-Luhn, et al. 2022).

## AI Evaluation

Consequently, I propose that the formal evaluation of healthcare AI should become the key area for attention. Both the WHO in its guidance (World Health Organization 2021) and GDPR in article 35 highlight the need for such processes. While the consent process ensures that the patient is aware of the risks associated with a particular care plan and ensures that their care is delivered in accordance with the values they espouse, it should not be used to allow healthcare organizations to provide defective care by shifting the blame for any potential mishaps to the patient— healthcare providers still have a responsibility to ensure the care is safe, that they are not in breach of duty, and that informed consent is not simply used as a shield to protect them from legal liability if something goes wrong when providing substandard AI-enhanced care. In principle, we evaluate the drugs, devices, and procedures we utilize in patient care, with regulators permitting some but not others or recommending these rather than those. Similarly, we should ensure that AI technologies are evaluated appropriately to ensure not only that they are clinically and financially effective but that they also meet good standards of privacy and security. This will ensure that patients can benefit from the aid of AI and that the use of standards for consent similar to those used in care involving drugs, medical devices, and other procedures is appropriate for AI-enhanced care. Patient consent should certainly not be used as a way to justify the use of poorly evaluated and monitored AI systems.

NICE has recently developed a framework for the evaluation of healthcare AI that makes reference to the ten guiding principles that can inform the development of good machine learning practice identified jointly by the relevant Canadian, U.K., and U.S. public bodies. While not mentioning “privacy” explicitly, it mentions data management and cybersecurity and addresses other points mentioned in this paper (FDA et al. 2021; NICE 2018). In England, digital technology assessment criteria (DTAC), as well as the DCB0129 and DCB0160 standards, have been brought forward to ensure that healthcare digital technologies (including AI applications) that are brought into clinical practice are evaluated from a clinical safety perspective, as well as from the point of data security and usability (NHS England n.d.; NHS England 2022a; NHS England 2022b).^7^ Additionally, current moves to treat some digital healthcare applications as medical devices (Medicines and Healthcare products Regulatory Agency (MHRA) 2022) opens the possibility to report healthcare AI-related incidents to the Yellow Card scheme (MHRA n.d.), which monitors adverse events related to medication and medical devices in a similar way to the U.S. Food and Drug Administration’s MAUDE database, and to which anyone can make a submission to raise concerns, allowing for ongoing monitoring of these technologies even when they have been cleared for release to the market. Finally, channels should be available so that patients can complain as easily about AI-enhanced care as they can about traditional care—for example, via patient liaisons or experience teams linked to their healthcare centres. The changing landscape of digital technology regulation and the emphasis on technology evaluation in the clinical informatics community (Friedman, et al. 2022) provide for a positive outlook for how AI-enhanced care will be implemented in the future and reassurance that key concerns regarding healthcare AI are becoming addressed.

Lastly, the burden imposed by GDPR on patient consent to AI-enhanced healthcare appears to be disproportionate in the long term if clinical AI becomes commonplace. While we first need to ensure that proper systems are in place to evaluate and monitor healthcare AI, as we do for healthcare professionals, medications, and medical devices, once this is achieved and AI becomes ubiquitous in healthcare, the demand of GDPR for explicit consent to AI-enhanced diagnosis and treatment may become overly burdensome, especially when considering the common law standard for informed consent. As already mentioned, if the way data is utilized in regular healthcare practice changes and AI involvement becomes the new normal, giving free consent to such care might become impossible according to GDPR and explicitly mentioning AI involvement in healthcare may become superfluous.

## Conclusion

I have argued here that while the adoption of AI is associated with concerns regarding privacy, reliability, and safety, these concerns are not new to healthcare. AI does not seem to provide any new types of challenges in the context of consent—all the issues affecting AI seem to be similar to those affecting traditional medicine, except we admit them more openly. While *Montgomery v Lanarkshire* provides the general standard for patient consent, GDPR provides another layer of obligation with respect to consent to AI-enhanced healthcare in relation to data. Nevertheless, these additional requirements may become superfluous once AI-enhanced healthcare becomes ubiquitous and well-regulated through the support of appropriate evaluation processes. This is not to say that the concept of GDPR is ill-conceived, but that its current requirements are not reasonable in the long term with respect to healthcare AI, as judged by current practices in medicine. As such, while AI might cause a paradigmatic shift in healthcare, it will not cause a paradigm shift for patient consent.

## Data Availability

Not applicable.

## Footnotes

1 The right to explanation is an important aspect of the debate at hand, and I make reference to it at various points throughout this text, but detailed discussion on it is beyond the scope of this paper. See the GDPR section for a brief discussion, nevertheless.

2 Regulation (EU) 2016/679 of the European Parliament and of the Council of 27 April 2016 on the protection of natural persons with regard to the processing of personal data and on the free movement of such data (United Kingdom General Data Protection Regulation). Available from legislation.gov.uk.

3 I use this phrase as the legislation was originally enacted in the European Union, but the United Kingdom kept it after Brexit via the European Union (Withdrawal) Act 2018 as amended by the Data Protection, Privacy and Electronic Communications (Amendments etc) (EU Exit) Regulations 2019 (SI 2019/419). As such, how it is phrased varies dependently on whether one reads the United Kingdom or European Union version of the GDPR.

4 Lorenzini et al. (2022 3) describe this as the problem of “ontology” arguing that if we treat AI as a tool then we do not need to disclose its use to the patient, while Cohen (2020, 1447; and Hodge 2021 follows this opinion) notes that if we regard AI as a “member of the care team” then we need to give the patient information about the AI’s “expertise.”

5 A limitation of this study is that it did not explore the views of patients on the issue of informed consent to healthcare AI, and only included the view of healthcare professionals/trainees and bioethicists.

6 Kamensky (2020) states that approximately 86 per cent of healthcare professionals use at least one type of AI in their practice, but it is unclear where this estimate is taken from, to what geographical region it applies, and what healthcare professionals it refers to.

7 For a review of EU legislation, see Schneeberger, et al. (2020). See also Kiseleva, et al. (2022).
